# Weight Loss and Usage of an Online Commercial Weight Loss Program (the CSIRO Total Wellbeing Diet Online) Delivered in an Everyday Context: Five-Year Evaluation in a Community Cohort

**DOI:** 10.2196/20981

**Published:** 2021-06-07

**Authors:** Gilly A Hendrie, Danielle L Baird, Emily Brindal, Gemma Williams, Jennie Brand-Miller, Beverly Muhlhausler

**Affiliations:** 1 Nutrition and Health Program Commonwealth Scientific and Industrial Research Organisation Adelaide Australia; 2 Charles Perkins Centre and School of Life and Environmental Sciences University of Sydney Sydney Australia

**Keywords:** obesity, obesity management, weight loss, internet-based intervention

## Abstract

**Background:**

Obesity is a global public health challenge, and there is a need for more evidence-based self-management programs that support longer-term, sustained weight loss.

**Objective:**

This study used data from the Commonwealth Scientific and Industrial Research Organisation (CSIRO) Total Wellbeing Diet Online program to determine the reach and weight loss results over its first 5 years.

**Methods:**

Participants were adults who joined the commercial weight loss program of their own volition between October 2014 and September 2019 (N=61,164). Information collected included year of birth, sex, height, weight, and usage data (eg, entries into the food diary, views of the menu, and program content). Weight loss and percentage of starting body weight lost were calculated. Members were divided into 2 groups for analysis: “stayers” were members who signed up for at least 12 weeks of the program and recorded a weight entry at baseline and at the end of the program, while “starters” began the program but did not record a weight after 12 weeks. Descriptive statistics and multiple linear regression were used to describe weight loss and determine the member and program characteristics associated with weight loss.

**Results:**

Data were available from 59,686 members for analysis. Members were predominately female (48,979/59,686, 82.06%) with an average age of 50 years (SD 12.6). The average starting weight was 90.2 kg (SD 19.7), and over half of all members (34,195/59,688, 57.29%) were classified as obese. At week 12, 94.56% (56,438/59,686) of the members had a paid program membership, which decreased to 41.48% (24,756/59,686) at 24 weeks. At week 12, 52.03% (29,115/55,958) of the remaining members were actively using the platform, and by week 24, 26.59% (14,880/55,958) were using the platform. The average weight loss for all members was 2.8 kg or 3.1% of their starting body weight. Stayers lost 4.9 kg (5.3% of starting body weight) compared to starters, who lost 1.6 kg (1.7% of starting body weight). Almost half (11,082/22,658, 48.91%) the members who stayed on the program lost 5% or more of their starting body weight, and 15.48% (3507/22,658) achieved a weight loss of 10% or more. Of the members who were classified as class 1 obese when they joined the program, 41.39% (3065/7405) who stayed on the program were no longer classified as obese at the end, and across all categories of obesity, 24% (3180/13,319) were no longer classified as obese at the end of the program. Based on multiple linear regression, platform usage was the strongest predictor of weight loss (β=.263; *P*<.001), with higher usage associated with greater weight loss.

**Conclusions:**

This comprehensive evaluation of a commercial, online weight loss program showed that it was effective for weight loss, particularly for members who finished the program and were active in using the platform and tools provided. If the results demonstrated here can be achieved at an even greater scale, the potential social and economic benefits will be extremely significant.

## Introduction

### Background

Obesity is a global public health challenge, with significant social and economic impacts. In Australia, two-thirds of adults are classified as overweight or obese [1], and this is expected to increase to more than three-quarters of the adult population by 2030 [2]. The costs to the Australian economy attributable to overweight status and obesity were estimated to be A $8.6 billion between 2011 and 2012 [1]. There is growing recognition that nutrition plays a crucial role in the etiology of chronic diseases and that chronic diseases such as type 2 diabetes can also be reversed by diet and lifestyle interventions [3]. With most adults struggling to control their weight, there is a need for more evidence-based self-management programs that support healthy diet and lifestyle patterns to promote longer-term, sustained weight loss [4].

In 2020, commercial weight loss services in Australia were estimated to be worth A $452 million [5]. Although there are several programs with a variety of dietary patterns, multiple literature reviews have concluded that weight loss results across programs are similar and that the vast majority produce only small to moderate effects which are not maintained in the longer term [6-8]. Reviews of branded and commercial programs have been primarily based on randomized controlled trials (RCTs) and suggest individuals who complete these programs can achieve significant weight loss [8]. However, in evaluations undertaken through longitudinal follow-up of participants, only a small proportion of the original number of individuals are included [9,10]; therefore, the findings are unlikely to be representative of the full participant population.

### Web-Based Delivery of Weight Loss Programs

In an attempt to increase the accessibility and success of commercial weight loss programs, a number of web-based programs have been developed. One early meta-analysis suggested that web-based interventions achieved similar weight loss to controls but that those with enhanced features could achieve greater weight loss than those providing education alone [11]. Some but not all more recent reviews suggest that web-based programs can be more successful than alternate delivery approaches, but the effect sizes are small, and the heterogeneity in study designs makes it difficult to determine the key elements driving weight loss [12-15].

Programs delivered online are growing in popularity and account for 17% of the market share for the diet industry [5]. Commercial programs that have traditionally relied on face-to-face group sessions have now shifted their focus to primarily digital delivery. An early review suggested that self-directed, online commercial programs were suboptimal [16], but since then, more advanced features have improved the offerings in the market, with associated RCTs suggesting moderate effects for weight loss. For example, one RCT that included 309 people on the 12-week Biggest Loser Club program reported weight losses of 2.0-3.2 kg compared to 0.5 kg in a wait-listed control group [17]. More recently, an evaluation of participants receiving the Weight Watchers Online program reported a 2.7 kg weight loss relative to a 1.3 kg loss in a control group receiving only a newsletter [18]. However, in a US study, participants on an eDiets program lost less weight over a 1-year period than did those receiving a comprehensive information manual (1.1% vs 4.0% of starting body weight, respectively). It should be noted that this evaluation was small and included only 47 participants in total [19]. Thus, based on the data from existing RCTs, it appears that online commercial weight loss programs can be successful. However, less has been published about how these programs may work in everyday contexts [16] where attrition is likely to be significant, especially when users are self-directed and incurring possible program costs.

In late 2014, the Commonwealth Scientific and Industrial Research Organisation (CSIRO) and Digital Wellness launched a commercial, online version of the CSIRO Total Wellbeing Diet. The dietary components were developed through clinical trials [20-23] and were initially translated into a series of popular books [24], estimated to have delivered weight loss benefits to 290,700 Australians, with an average weight loss of 5.7 kg [25]. The online format delivered the same program as the books did but through a digital platform, allowing for several enhancements, including personalized eating plans, customized weekly meal plans, food and exercise diaries, the ability to record and see progress of weight loss, a member forum, and supportive correspondence via email. Our study analyzed the data from the CSIRO Total Wellbeing Diet Online program in the first 5 years after it launched to determine the program’s reach and weight loss results over this period and to investigate the key determinants of weight loss success. Specifically, we aimed to determine average weight loss and its relationship to the duration of membership, features of the platform that members used most, and the member and usage characteristics that were associated with greater weight loss.

## Methods

### Study Design and Participants

Participants were individuals aged ≥18 years who joined the CSIRO Total Wellbeing Diet Online of their own volition between October 2014 and September 2019 (N=61,164; referred to as “members”). In the registration and setup processes, individuals younger than 18 years or who had a BMI that placed them in the underweight category (BMI <18.5 kg/m^2^) were automatically excluded. The participants who were removed from this analysis were as follows: pseudomembers (ie, platform testers or affiliated staff), those whose membership was paid for by their employer because their motivations might have been different to those who signed up and paid for their membership voluntarily, members living outside of Australia because the context in which they were following the program was different and the menu plans were not optimized for seasonal or local produce, and members without a paid subscription. These combined exclusions resulted in 1478 records being removed (1478/61,164; 2.4% of the available data).

### Intervention

The CSIRO Total Wellbeing Diet Online [26] is a 12-week higher-protein, lower–glycemic index, commercial weight loss program managed by Digital Wellness and available to individuals at a cost of A $199 for the first 12 weeks. The user registration process collects information on year of birth, sex, physical activity levels, and weight loss goals to tailor eating and exercise plans. Individuals are assigned to 1 of 3 kilojoule bands depending on their starting weight (6000, 7000, or 8000 kJ/day). The diet is structured around 3 meals (breakfast, lunch, and dinner) and 2 snacks each day through use of a food group system where portions of food are presented as standard units for each food group. There are 7 food groups: fruit, vegetables, meat and alternatives, breads and cereals, dairy, healthy fats and oils, and indulgences. Meals are designed around a template of standard units, which ensures daily allowances of food groups are met and provides optimal nutrition and energy to promote weight loss. Daily and weekly meal plans can be customized by swapping meals using the recipe database.

**Figure 1:**
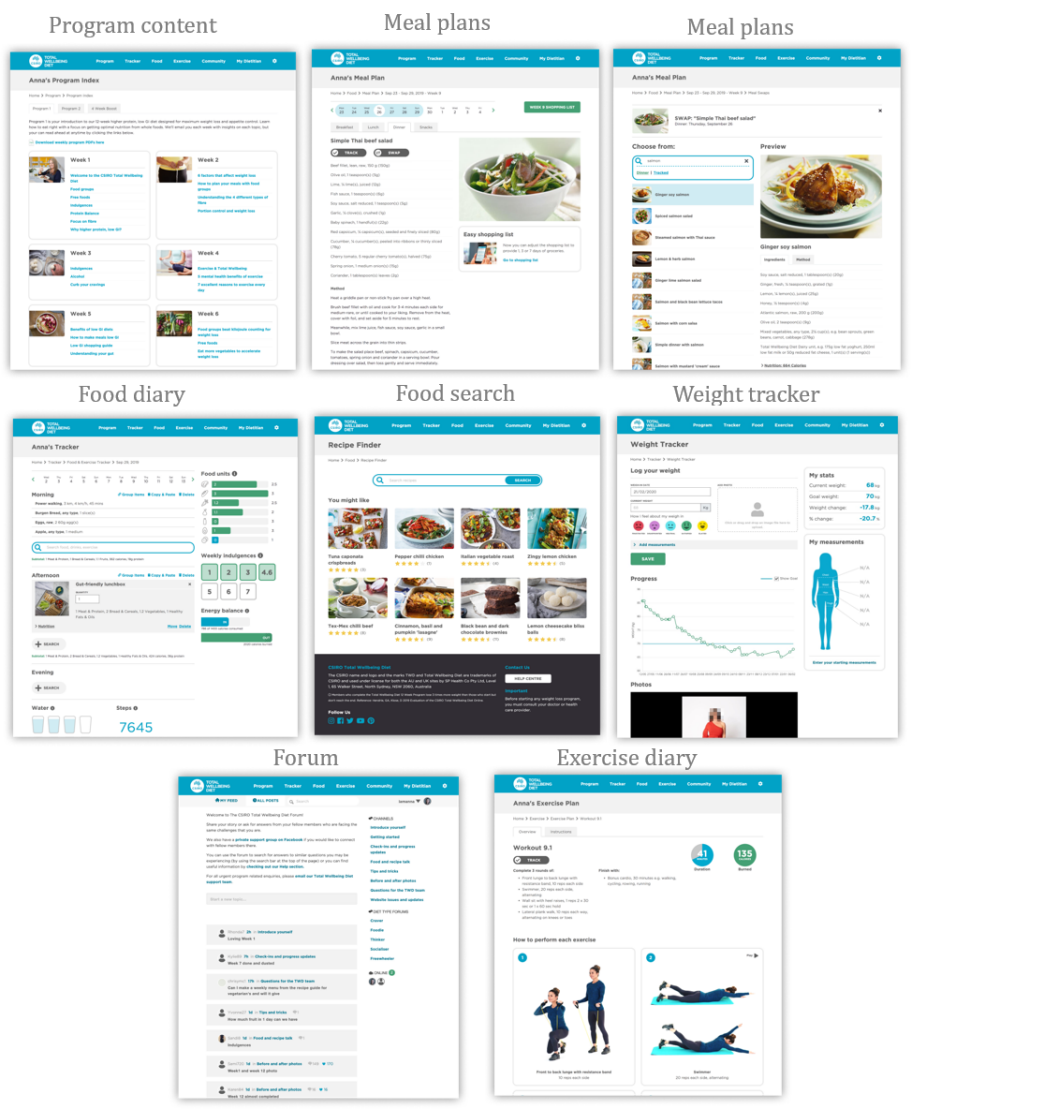
Screenshots of the features of the CSIRO Total Wellbeing Diet Online platform.

The online platform is a fully responsive web app with an interface that is optimized for viewing on a desktop and on mobile devices. The platform features are designed to support individuals throughout the program (Figure 1). Program content provides general information, nutrition advice, weekly tutorials available for viewing at any time, and some content sent out to members in a weekly email. The meal plans provide individuals with meals and snacks planned for the day or week ahead. The food diary can be used to log meals and snacks consumed, either by entering prepopulated recipes from the meal plan or recipe database, or by entering individual foods from a comprehensive food database. The food search function allows members to search through the food database for individual foods or recipes and view information about their composition and food units. The food tracker tallies the food units and total energy consumed over the day. The exercise diary is where completed exercises can be logged or where activities chosen from a database of different exercise types and intensities can be recorded. Members receive weekly emails on their nominated weigh-in day to remind them to weigh themselves and record their weight into the weight tracker. Progress data are presented in a graph and table form. The platform also has a forum for members to share their stories or discuss relevant issues with other members.

### Data Collection and Study Outcomes

Digital Wellness manages the collection and storage of data, including registration details and all user activity and interactions with the platform. In addition to information collected at registration, individuals’ body measurements, such as height, weight, and waist circumference, are also collected. Other data collected included program details (eg, paid membership duration) and platform usage data, including entries into the food diary, views of the menu plans, views of exercise plans, views of program content information, forum visits, searches of the food database, and weight entries. These data were provided to the research team in a deidentified format with each individual member assigned a unique identifier. As part of registration, participants agreed to their data being used for research purposes; therefore, no direct participant consent was sought. Ethics approval to conduct this research was received from the CSIRO Health and Medical Human Research Ethics Committee (approval #2019_090_LR).

Weight loss was calculated as the difference in kilograms between the last and first weight entered into the platform, with a larger number representing greater weight loss. Weight loss was also calculated as a percentage of starting body weight and was categorized into 4 groups: weight gain (greater than 0% difference), a weight loss of over 0% and less than 5% of starting body weight, 5% to less than 10% of starting body weight, and greater than or equal to 10% of starting body weight lost.

The date of program setup and total duration of paid membership were used to calculate the number of participants remaining in the program within the first 24 weeks from registration. Once paid membership duration lapsed, a member was considered to have left the program (dropped out of the program). Platform usage attrition within the first 24 weeks from registration was also determined by calculating the difference in days between the date of last user activity and the date of the program setup.

Platform usage was considered to be any logged activity and was assessed as usage of each of the 7 features and as overall usage (all features combined). Platform usage was described in 3 ways: “total days active” referred to the number of unique days a member used the platform irrespective of whether usage occurred multiple times per day, “percent active days” was calculated as total days active divided by the number of days of membership expressed as a percentage, and “activity per day” was calculated as the total user activity divided by the number of days of membership. Levels of platform usage were then calculated by creating quintiles (5 groups) based on activity per day, where quintile 1 represented the lowest platform activity and quintile 5 the highest platform activity. There was a small group of participants with very high use, and thus the creation of quintiles helped to manage this skewness.

### Statistical Analysis

All data were inspected for invalid records through a systematic and previously used data cleaning process based on erroneous height (less than 1 m or greater than 3 m) or weight values (less than 13 kg or greater than 250 kg), and extreme BMI values (less than 13 kg/m^2^ or greater than 97 kg/m^2^). Members were removed from analysis if a total weight loss could not be calculated (n=1451) due to no starting weight reported or only a single weight entry being made. Members were also removed from analysis if their weight value was the contributing factor to an invalid BMI (n=14) or if age at registration was calculated from entered data as less than 18 years or above 100 years of age (n=7). In addition, there were 6 pseudomembers removed from analysis. After these exclusions, 59,686 members were included in this analysis (59,686/61,164, 97.58% of all members).

Members were divided into 2 groups for analysis: *“*stayers” were defined as members who signed up to at least 12 weeks of the program and entered their weight into the platform at baseline and at the end of the program. This was calculated as members with a paid membership equal to or greater than 84 days (12-week program duration) and for whom days between their first and last entered weight were equal to or greater than 77 days (plus or minus 1 week for first or last weigh-in). There were 22,658 members who were categorized as stayers, and the average time between first and last weigh-in was 268 days. Starters were defined as members who started the program and had some level of engagement with the platform but did not enter a weight at the end of the 12-week program. By definition, starters had a shorter duration of paid membership or a last-entered weight that was before 77 days after baseline. There were 37,028 members who were categorized as starters with an average of 23 days between the first and last weigh-in.

The average (and SD) weight loss and percentage weight loss were calculated at a group level for all members and by subgroups of interest. These subgroups included those of sex, age (19-30 years, 31-50 years, 51-70 years, and 70 years and over), starting BMI category (normal weight, overweight, obese class 1, obese class 2, and obese class 3), and socioeconomic status according to quintiles of Socio-Economic Indexes for Areas (SEIFA), where a lower quintile represents a greater disadvantage [27]. Differences in weight loss, percentage weight loss, and platform usage were examined between subgroups of members, with significance being tested by 1-way analysis of variance (ANOVA).

Multiple linear regression was used to assess which member characteristics (sex, age, SEIFA quintile, starting BMI) or program characteristics (membership length, activity per day) were the strongest predictors of weight loss. The primary analysis used the last weight entered into the platform (ie, the last observation carried forward). The models were run to predict total weight loss on the program (using total weight loss in kilograms and as a percentage of starting body weight). Member and program characteristics (predictors) were added to the model simultaneously. A secondary sensitivity analysis was conducted to determine the robustness of the primary results with baseline weight carried forward being used when participants did not have a weight value available in the platform at 12 weeks or beyond. The regression models were run for all members and by subgroup for starters and stayers separately. The significant predictors of weight loss were similar to those used for total weight loss and percentage of body weight lost; therefore, only the regression results for all members predicting percentage of body weight lost are presented. Statistical analyses were performed using SPSS Statistics 25 (IBM Corp).

## Results

### Characteristics of Members

Members who signed up to the program were predominately female (48,979/59,686; 82.06%) with an average age of 50 years (SD 12.6). Overall, 40.16% (23,969/59,686) of members were aged between 31-50 years, and 48.70% (29,068/59,686) were between 51-70 years (Table 1). The average starting weight of members when they joined the program was 90.2 kg (SD 19.7), the average BMI was 32.2 (SD 6.3; Table S1, Multimedia Appendix 1), and over half of all members were classified as obese (34,195/59,686, 57.29%). More specifically, 30.75% (18,353/59,686) of members were classified as class 1 obese, 16.11% (9613/59,686) as class 2, and 10.44% (6229/59,686) as class 3, the highest risk group (Table 1). Men were heavier than women (104.9 kg vs 87.0 kg, respectively), but the starting BMI of men and women was similar (32.8 vs 32.0, respectively; Table S1, Multimedia Appendix 1).

Members resided in all states of Australia (data not shown). One-quarter (15,005/59,686, 25.14%) of members lived in areas classified in the lowest 2 quintiles of disadvantage (most disadvantaged), and 32.84% (19,598/59,686) lived in areas classified as the least disadvantaged (Table 1). Members living in the most disadvantaged areas were heavier (94.1 kg vs 87.7 kg) and had a higher BMI (33.9 vs 31.1) compared to those in the least disadvantaged areas (Table S1, Multimedia Appendix 1).

**Table 1:** Demographic characteristics of starters (n=37,028), stayers (n=22,658), and all members (N=59,686) at the time of joining the CSIRO Total Wellbeing Diet Online.

Member characteristics					Starters, n (%)			Stayers, n (%)			All members, n (%)
**Sex**											
	Male			6204 (16.75)			4503 (19.87)			10,707 (17.94)	
	Female			30,824 (83.25)			18,155 (80.13)			48,979 (82.06)	
**Age (years)**											
	18-30			2312 (6.24)			1004 (4.43)			3316 (5.56)	
	31-50			15,766 (42.58)			8203 (36.20)			23,969 (40.16)	
	51-70			17,108 (46.20)			11,960 (52.78)			29,068 (48.70)	
	>71			1842 (4.97)			1491 (6.58)			3333 (5.58)	
**Starting BMI category**											
	Normal weight			3771 (10.19)			1473 (6.51)			5244 (8.79)	
	Overweight			11,171 (30.19)			7786 (34.39)			18,957 (31.78)	
	**Obese**			20,859 (56.37)			13,336 (58.90)			34,195 (57.33)	
		Class 1	10,937 (29.55)			7416 (32.75)			18,353 (30.77)		
		Class 2	5956 (16.09)			3657 (16.15)			9613 (16.12)		
		Class 3	3966 (10.72)			2263 (10.00)			6229 (10.44)		
	Missing/invalid			1205 (3.26)			46 (0.20)			1251 (2.10)	
**Socioeconomic status**											
	1 (lowest)			3665 (9.90)			2322 (10.25)			5987 (10.03)	
	2			5579 (15.07)			3439 (15.18)			9018 (15.11)	
	3			6971 (18.83)			4446 (19.62)			11,417 (19.13)	
	4			8032 (21.69)			4894 (21.60)			12,926 (21.66)	
	5 (highest)			12,251 (33.09)			7347 (32.43)			19,598 (32.84)	
	Unknown			530 (1.43)			210 (0.93)			740 (1.24)	

### Membership and Platform Attrition

**Figure 2:**
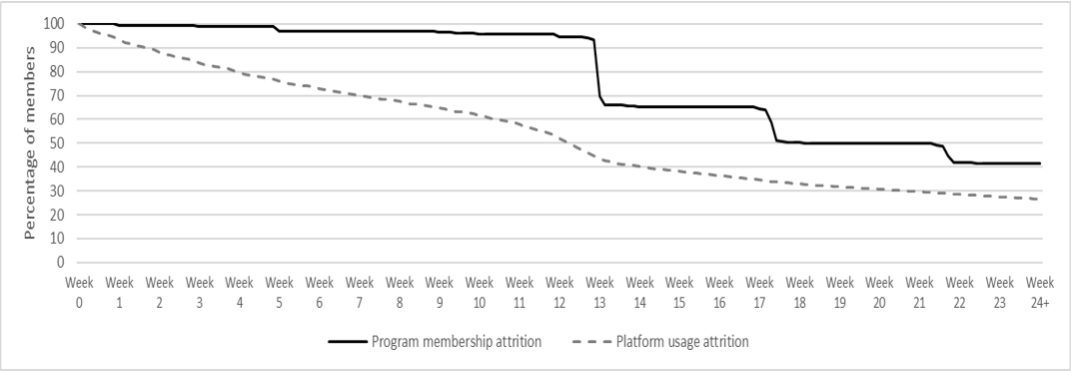
Program membership and platform usage attrition over 24 weeks for members of the CSIRO Total Wellbeing Diet Online (n=59,686).

The percentage of members with paid membership and active platform usage declined gradually over the first 24 weeks (Figure 2). At week 12, 94.56% (56,438/59,686) of members had a paid membership, and at week 13, this decreased to 69.65% (41,573/59,686) of members. By week 14, 65.34% (39,000/59,686) of the original membership base had a paid membership, which continued to decrease to 41.48% (24,756/59,686) of members at 24 weeks. In terms of platform usage, 83.80% (46,893/55,958) of members were using the platform (that is any logged data recorded) after 3 weeks, 52.03% (29,115/55,958) of remaining members were using the platform at week 12, and 26.59% (14,880/55,958) of those remaining were still using the platform by week 24 (Figure 2).

### Weight Loss

The primary analysis using last observation carried forward indicated an average weight loss for all members of 2.8 kg or 3.1% of starting body weight. Stayers lost an average of 4.9 kg (5.3% of starting body weight) compared to starters, who lost an average of 1.6 kg (1.7% of starting body weight). The starting BMI of members classified as starters and stayers was similar to that at the start of the program. Stayers who were classified as overweight when they joined the program lost 4.9% of their starting body weight, compared to 5.5% for those classified as class 1 obese, 5.6% as class 2, and 5.9% as class 3. Stayers living in the most disadvantaged areas lost 5.8% of their starting body weight compared to 4.9% for those in areas of least disadvantage (Table 2).

Overall, 24.37% (14,546/59,686) of all members achieved a weight loss of 5% or more of their starting body weight. Almost half (11,082/22,658, 48.91%) of the stayers lost 5% or more of their starting body weight compared to 9.36% (3464/37,028) of starters (Table 3). Among stayers, 33.43% (7575/22,658) achieved a weight loss of 5% to <10%, and 15.48% (3507/22,658) achieved a weight loss of 10% or more of their starting body weight. The proportion of members losing >10% of their starting weight was highest for males (lost 10% or more of their starting body weight: 1012/4503, 22.47%), members classified as class 3 obese at baseline (445/2263, 19.66%), and those living in the most disadvantaged areas (409/2322, 17.61%; Table 3).

Table 4 shows the weight status of members at the time they joined and at the end of the program. Overall, 14.27% (2701/18,930) of all members who were classified as overweight when they joined the program had achieved normal weight by the end. Among stayers, this proportion was 24.59% (1911/7770). Across all levels of obesity, 24% (3180/13,319) of stayers who were classified as obese when they joined the program were no longer classified as obese at the end. More specifically, among stayers who were classified as class 1 obese at baseline, 41.39% (3065/7405) had shifted to a lower weight status category at the end of the program (ie, overweight or normal weight). In addition, 47.86% (1748/3652) and 36.78% (832/2262) of stayers who were classified as class 2 and 3, respectively, achieved a lower weight status at the end of the program.

**Table 2:** Weight loss in kilograms and percentage of body weighta in starters (n=37,028), stayers (n=22,658), and all members (n=59,686) on the CSIRO Total Wellbeing Diet Online.

Member characteristics			Starters, mean (SD)		Stayers, mean (SD)		All members, mean (SD)	
			Weight loss (kg)	% body weight	Weight loss (kg)	% body weight	Weight loss (kg)	% body weight
Total			1.60 (3.51)	1.73 (3.36)	4.87 (5.60)	5.25 (5.56)	2.84 (4.69)	3.07 (4.65)
**Sex**								
	Male		2.28 (4.44)	2.14 (3.79)	6.85 (6.26)	6.51 (5.45)	4.20 (5.75)	3.98 (5.05)
	Female		1.47 (3.27)	1.65 (3.26)	4.38 (5.31)	4.94 (5.54)	2.55 (4.38)	2.87 (4.54)
**Age (years)**								
	18-30		1.64 (2.70)	1.80 (2.99)	4.48 (6.22)	4.94 (6.29)	2.50 (4.30)	2.75 (4.50)
	31-50		1.61 (3.24)	1.72 (3.18)	4.46 (5.70)	4.75 (5.63)	2.58 (4.46)	2.76 (4.42)
	51-70		1.61 (3.71)	1.75 (3.41)	5.12 (5.56)	5.54 (5.51)	3.06 (4.88)	3.31 (4.78)
	>71		1.43 (4.55)	1.54 (4.59)	5.39 (4.61)	5.99 (4.76)	3.20 (4.98)	3.53 (5.16)
**Starting BMI category**								
	Normal weight		0.76 (2.41)	1.15 (3.90)	2.38 (3.03)	3.58 (4.51)	1.22 (2.70)	1.83 (4.23)
	Overweight		1.40 (2.71)	1.78 (3.51)	3.87 (4.05)	4.91 (5.11)	2.42 (3.54)	3.07 (4.51)
	**Obese**		1.92 (4.00)	1.88 (3.19)	5.72 (6.31)	5.66 (5.84)	3.41 (5. 36)	3.34 (4.78)
		Class 1	1.75 (2.54)	1.90 (2.27)	5.11 (5.31)	5.54 (5.63)	3.11 (4.24)	3.37 (4.52)
		Class 2	1.93 (3.14)	1.84 (3.01)	5.90 (6.16)	5.64 (5.80)	3.44 (4.93)	3.29 (4.67)
		Class 3	2.41 (7.14)	1.85 (4.42)	7.45 (8.77)	5.94 (6.55)	4.24 (8.14)	3.34 (5.65)
	Missing/invalid		0.56 (3.15)	0.56 (2.40)	7.61 (14.70)	7.53 (8.81)	0.82 (4.37)	0.82 (3.17)
**Socioeconomic status**								
	1 (lowest)		1.78 (4.77)	1.82 (4.13)	5.58 (6.10)	5.80 (5.76)	3.26 (5.64)	3.36 (5.20)
	2		1.71 (2.68)	1.83 (2.58)	5.20 (5.77)	5.53 (5.56)	3.04 (4.47)	3.24 (4.37)
	3		1.70 (4.14)	1.81 (3.99)	5.00 (5.46)	5.34 (5.58)	2.98 (4.97)	3.19 (4.98)
	4		1.59 (3.71)	1.72 (3.58)	4.97 (5.56)	5.37 (5.50)	2.87 (4.79)	3.10 (4.75)
	5 (highest)		1.49 (2.85)	1.66 (2.89)	4.39 (5.27)	4.87 (5.40)	2.57 (4.18)	2.86 (4.31)
	Unknown		0.83 (1.66)	0.92 (1.83)	3.49 (9.02)	3.81 (8.15)	1.59 (5.14)	1.74 (4.78)

^a^Average weight loss in kilograms and percentage of starting body weight were calculated using last observation carried forward.

**Table 3:** Percentage of the sample within weight loss categoriesa,b for starters (n=37,028), stayers (n=22,658), and all members (N=59,686) on the CSIRO Total Wellbeing Diet Online.

Member characteristics					Starters, n (%)													Stayers, n (%)													All members, n (%)									
					Weight gain			0%-<5% lost			5%-<10% lost			≥10% lost			Weight gain				0%-<5% lost			5%-<10% lost			≥10% lost			Weight gain				0%-<5% lost			5%-<10% lost			≥10% lost
Total					2227 (6.01)			31337 (84.63)			3148 (8.5)			316 (0.85)			2518 (11.11)				9058 (39.98)			7575 (33.43)			3507 (15.48)			4745 (7.95)				40395 (67.68)			10723 (17.97)			3823 (6.41)
**Sex**																																								
	Male			284 (4.58)			5006 (80.69)			815 (13.14)			99 (1.6)			317 (7.04)				1489 (33.07)			1685 (37.42)			1012 (22.47)			601 (5.61)				6495 (60.66)			2500 (23.35)			1111 (10.38)	
	Female			1943 (6.3)			26331 (85.42)			2333 (7.57)			217 (0.7)			2201 (12.12)				7569 (41.69)			5890 (32.44)			2495 (13.74)			4144 (8.46)				33900 (69.21)			8223 (16.79)			2712 (5.54)	
**Age (years)**																																								
	18-30			160 (6.92)			1896 (82.01)			219 (9.47)			37 (1.6)			132 (13.15)				377 (37.55)			349 (34.76)			146 (14.54)			292 (8.81)				2273 (68.55)			568 (17.13)			183 (5.52)	
	31-50			1055 (6.69)			13287 (84.28)			1294 (8.21)			130 (0.82)			1136 (13.85)				3359 (40.95)			2584 (31.5)			1124 (13.7)			2191 (9.14)				16646 (69.45)			3878 (16.18)			1254 (5.23)	
	51-70			919 (5.37)			14558 (85.09)			1497 (8.75)			134 (0.78)			1158 (9.68)				4741 (39.64)			4081 (34.12)			1980 (16.56)			2077 (7.15)				19299 (66.39)			5578 (19.19)			2114 (7.27)	
	71 +			93 (5.05)			1596 (86.64)			138 (7.49)			15 (0.81)			92 (6.17)				581 (38.97)			561 (37.63)			257 (17.24)			185 (5.55)				2177 (65.32)			699 (20.97)			272 (8.16)	
**Starting BMI category**																																								
	Normal weight			252 (6.68)			3280 (86.98)			227 (6.02)			12 (0.32)			226 (15.34)				732 (49.69)			427 (28.99)			88 (5.97)			478 (9.12)				4012 (76.51)			654 (12.47)			100 (1.91)	
	Overweight			710 (6.36)			9374 (83.91)			987 (8.84)			100 (0.9)			895 (11.49)				3162 (40.61)			2682 (34.45)			1047 (13.45)			1605 (8.47)				12536 (66.13)			3669 (19.35)			1147 (6.05)	
	**Obese**			1238 (5.94)			17529 (84.04)			1896 (9.09)			196 (0.94)			1391 (10.43)				5140 (38.54)			4444 (33.32)			2361 (17.70)			2629 (7.69)				22669 (66.29)			6340 (18.54)			2557 (7.48)	
		Class 1	676 (6.18)			9087 (83.08)			1073 (9.81)			101 (0.92)			804 (10.84)				2797 (37.72)			2511 (33.86)			1304 (17.58)			1480 (8.06)				11884 (64.75)			3584 (19.53)			1405 (7.66)		
		Class 2	359 (6.03)			5012 (84.15)			534 (8.97)			51 (0.86)			374 (10.23)				1434 (39.21)			1237 (33.83)			612 (16.74)			733 (7.63)				6446 (67.06)			1771 (18.42)			663 (6.90)		
		Class 3	203 (5.12)			3430 (86.49)			289 (7.29)			44 (1.11)			213 (9.41)				909 (40.17)			696 (30.76)			445 (19.66)			416 (6.68)				4339 (69.66)			985 (15.81)			489 (7.85)		
	Missing /invalid			24 (1.99)			1136 (94.27)			37 (3.07)			8 (0.66)			2 (4.35)				17 (36.96)			17 (36.96)			10 (21.74)			26 (2.08)				1153 (92.17)			54 (4.32)			18 (1.44)	
**Socioeconomic status**																																								
	1 (lowest)			222 (6.06)			3055 (83.36)			351 (9.58)			37 (1.01)			226 (9.73)				874 (37.64)			813 (35.01)			409 (17.61)			448 (7.48)				3929 (65.63)			1164 (19.44)			446 (7.45)	
	2			312 (5.59)			4728 (84.75)			496 (8.89)			43 (0.77)			346 (10.06)				1310 (38.09)			1214 (35.3)			569 (16.55)			658 (7.3)				6038 (66.95)			1710 (18.96)			612 (6.79)	
	3			405 (5.81)			5876 (84.29)			619 (8.88)			71 (1.02)			474 (10.66)				1729 (38.89)			1520 (34.19)			723 (16.26)			879 (7.7)				7605 (66.61)			2139 (18.74)			794 (6.95)	
	4			502 (6.25)			6821 (84.92)			647 (8.06)			62 (0.77)			537 (10.97)				1904 (38.9)			1673 (34.18)			780 (15.94)			1039 (8.04)				8725 (67.5)			2320 (17.95)			842 (6.51)	
	5 (highest)			762 (6.22)			10376 (84.7)			1011 (8.25)			102 (0.83)			899 (12.24)				3146 (42.82)			2300 (31.31)			1002 (13.64)			1661 (8.48)				13522 (69)			3311 (16.89)			1104 (5.63)	
	Unknown			24 (4.53)			481 (90.75)			24 (4.53)			1 (0.19)			36 (17.1)				95 (45.2)			55 (26.2)			24 (11.4)			60 (8.11)				576 (77.84)			79 (10.68)			25 (3.38)	

^a^Average weight loss in kilograms and percentage of starting body weight were calculated using last observation carried forward.

^b^Proportion of the sample within each category of weight loss: weight gain, 0 to less than 5% of starting body weight lost, 5% to less than 10% of starting body weight lost, and greater than or equal to 10% of starting body weight lost.

**Table 4:** Shift in body weight status category as a percentage of starting weight status category for starters (n=37,028), stayers (n=22,658), and all members (N=59,686) in the CSIRO Total Wellbeing Diet Online.

Starting weight status category		Final weight status category, n (%)				
		Normal weight	Overweight	Class 1 obese	Class 2 obese	Class 3 obese
**Starters**						
	Normal weight (n=3767)	3716 (98.65)	45 (1.19)	4 (0.11)	1 (0.03)	1 (0.03)
	Overweight (n=11,160)	790 (7.08)	10,297 (92.27)	68 (0.61)	1 (0.01)	4 (0.04)
	Class 1 obese (n=10,933)	9 (0.08)	1572 (14.38)	9286 (84.94)	65 (0.59)	1 (0.01)
	Class 2 obese (n=5955)	4 (0.07)	5 (0.08)	1053 (17.68)	4871 (81.80)	22 (0.37)
	Class 3 obese (n=3966)	5 (0.13)	6 (0.15)	7 (0.18)	502 (12.66)	3446 (86.89)
**Stayers**						
	Normal weight (n=1468)	1391 (94.75)	76 (5.18)	1 (0.07)	0 (0.00)	0 (0.00)
	Overweight (n=7770)	1911 (24.59)	5688 (73.20)	166 (2.14)	2 (0.03)	3 (0.04)
	Class 1 obese (n=7405)	56 (0.76)	3009 (40.63)	4223 (57.03)	113 (1.53)	4 (0.05)
	Class 2 obese (n=3652)	11 (0.30)	92 (2.52)	1645(45.04)	1851 (50.71)	52 (1.42)
	Class 3 obese (n=2262)	4 (0.18)	8 (0.35)	64 (2.83)	756 (33.42)	1430 (63.22)
**All members**						
	Normal weight (n=5235)	5107 (97.55)	121 (2.31)	5 (0.10)	1 0.02)	1 (0.02)
	Overweight (n=18,930)	2701 (14.27)	15985 (84.44)	234 (1.24)	3 (0.02)	7 (0.04)
	Class 1 obese (n=18,338)	65 (0.35)	4581 (24.98)	13,509 (73.67)	178 (0.97)	5 (0.03)
	Class 2 obese (n=9607)	15 (0.16)	97 (1.01)	2698 (28.08)	6723 (69.98)	74 (0.77)
	Class 3 obese (n=6228)	9 (0.14)	14 (0.22)	71 (1.14)	1258 (20.20)	4876 (78.29)

### Platform Usage

Overall members used the platform on 29.8% of their membership days, and this was higher in stayers than starters (46.5% vs 19.6%, respectively). The most commonly used platform features were the weigh-in (used by all members), food diary (52,828/59,686, 88.51% of members), and menu plans (51,718/59,686, 86.65% of members). The total number of days (irrespective of membership length) members were active on the features was highest for the food diary (15.10 days), menu plans (13.17 days), and weigh-in feature (12.27 days), but activity per day of membership was highest for the food diary and menu plan (used approximately 2 out of every 3 days; Table 5).

The usage per day of membership was higher in stayers for all features. On average, stayers used the menu plans and food diary once per day, while starters used these less than once every 2 days. Stayers used the weigh-in once per week, while starters used this feature less than once every fortnight (Table 5).

**Table 5:** Platform feature usage for starters (n=37,028), stayers (n=22,658) and all members (n=59,686) of the CSIRO Total Wellbeing Diet Online.

Platform feature		Usage, n (%)^a^	Total days active, mean (SD)^b^	Active days, mean (SD)^c^	Activity per day, mean (SD)^d^
**Starters (n=** **37,028)**					
	Overall	37,028 (100.00)	22.02 (24.34)	19.61 (28.70)	0.98 (2.09)
	Weigh-in	36,965 (99.83)	4.63 (4.98)	4.30 (8.50)	0.04 (0.08)
	Food diary	30,900 (83.45)	6.76 (9.27)	6.05 (10.30)	0.37 (0.89)
	Menu plan	30,637 (82.74)	6.73 (10.40)	5.70 (9.92)	0.48 (1.17)
	Exercise plan	19,654 (53.08)	1.35 (2.58)	1.25 (2.87)	0.03 (0.07)
	Program content	17,203 (46.46)	1.01 (1.93)	0.92 (2.18)	0.01 (0.03)
	Forum	13,319 (35.97)	0.89 (2.46)	0.83 (2.94)	0.02 (0.07)
	Food search	9538 (25.76)	0.63 (1.99)	0.57 (2.69)	0.03 (0.44)
**Stayers (n=22,658)**					
	Overall	22,658 (100.00)	90.89 (113.54)	46.50 (46.59)	2.30 (3.12)
	Weigh-in	22,615 (99.81)	24.75 (35.18)	12.79 (18.44)	0.13 (0.18)
	Food diary	21,926 (96.77)	28.73 (45.73)	14.97 (18.21)	0.95 (1.58)
	Menu plan	21,079 (93.03)	23.69 (39.93)	11.58 (15.16)	1.03 (1.71)
	Exercise plan	15,992 (70.58)	3.77 (8.40)	2.14 (4.84)	0.05 (0.13)
	Program content	16,021 (70.71)	2.89 (5.69)	1.62 (3.00)	0.02 (0.06)
	Forum	12,679 (55.96)	4.14 (19.89)	1.99 (6.25)	0.04 (0.24)
	Food search	11,372 (50.19)	2.92 (9.21)	1.41 (3.66)	0.08 (0.31)
**All members (n=59,686)**					
	Overall	59,686 (100.00)	48.17 (79.86)	29.82 (38.80)	1.48 (2.61)
	Weigh-in	59,579 (99.82)	12.27 (24.09)	7.52 (13.82)	0.08 (0.14)
	Food diary	52,828 (88.51)	15.10 (31.00)	9.43 (14.51)	0.59 (1.23)
	Menu plan	51,718 (86.65)	13.17 (27.21)	7.93 (12.50)	0.69 (1.43)
	Exercise plan	35,650 (59.73)	2.27 (5.69)	1.59 (3.77)	0.03 (0.1)
	Program content	33,227 (55.67)	1.73 (3.93)	1.18 (2.54)	0.02 (0.05)
	Forum	25,999 (43.56)	2.13 (12.50)	1.27 (4.53)	0.03 (0.16)
	Food search	20,914 (35.04)	1.50 (5.99)	0.89 (3.12)	0.05 (0.40)

^a^Percentage of the sample who used the platform or feature at any time during their membership.

^b^Total days active refers to the number of unique days a member used the platform irrespective of whether usage occurred multiple times per day.

^c^Percent active days was calculated as total days active divided by number of days of membership expressed as a percentage.

^d^Activity per day was calculated as the total user activity divided by the number of days of membership.

### Weight Loss Across Levels of Platform Usage

Table 6 shows the average weight loss by levels of platform usage. High usage was characterized as viewing the menu plans 2-3 times per day, making 2 food diary entries per day, searching for foods once per week, recording a weight about once per week, and viewing the exercise plans and the forum a little less than once per week. Weight loss in kilograms and percentage body weight increased with increasing levels of platform usage. Overall, members with the highest usage lost 5.3% of their starting body weight compared to 0.9% for those with the lowest usage. Stayers who used the platform most lost 6.6% of their starting body weight compared to 3.0% for those with the lowest usage. Among stayers, 1 in 5 in the highest category of usage lost 10% or more of their starting body weight and 2 in 5 lost 5%-<10% of their starting body weight.

**Table 6:** Weight loss by level of platform usage for starters (n=37,028), stayers (n=22,658), and all members (N=59,686) in the CSIRO Total Wellbeing Diet Online.

Levels of platform usage			Weight loss, mean (SD)^a^					Weight loss in categories, n (%)^b^						
			Weight, kg		% body weight		Weight gain			0%-<5% lost		5%-<10% lost		≥10% lost
**Starters**														
	Quintile 1: lowest usage (n=10,471)	0.55 (2.78)		0.57 (3.12)		582 (5.56)			9610 (91.78)		237 (2.26)		42 (0.40)	
	Quintile 2 (n=8337)	1.40 (4.20)		1.45 (3.74)		627 (7.52)			7151 (85.77)		501 (6.01)		58 (0.70)	
	Quintile 3 (n=7290)	1.86 (3.75)		2.01 (3.28)		496 (6.80)			6047 (82.95)		677 (9.29)		70 (0.96)	
	Quintile 4 (n=6398)	2.31 (3.04)		2.54 (2.84)		357 (5.58)			5162 (80.68)		816 (12.75)		63 (0.98)	
	Quintile 5: highest usage (n=4532)	2.99 (3.05)		3.33 (2.91)		165 (3.64)			3367 (74.29)		917 (20.23)		83 (1.83)	
**Stayers**														
	Quintile 1: lowest usage (n=1466)	2.88 (6.15)		2.98 (6.30)		360 (24.56)			670 (45.70)		299 (20.40)		137 (9.35)	
	Quintile 2 (n=3600)	4.03 (5.86)		4.26 (5.70)		572 (15.89)			1583 (43.97)		987 (27.42)		458 (12.72)	
	Quintile 3 (n=4649)	4.45 (5.49)		4.72 (5.45)		606 (13.04)			2012 (43.28)		1436 (30.89)		595 (12.80)	
	Quintile 4 (n=5539)	4.82 (5.30)		5.21 (5.37)		591 (10.67)			2284 (41.23)		1853 (33.45)		811 (14.64)	
	Quintile 5: highest usage (n=7404)	5.98 (5.41)		6.56 (5.22)		389 (5.25)			2509 (33.89)		3000 (40.52)		1506 (20.34)	
**All members**														
	Quintile 1: lowest usage (n=11,937)	0.84 (3.46)		0.87 (3.75)		942 (7.89)			10280 (86.12)		536 (4.49)		179 (1.50)	
	Quintile 2 (n=11,937)	2.19 (4.91)		2.30 (4.61)		1199 (10.04)			8734 (73.17)		1488 (12.47)		516 (4.32)	
	Quintile 3 (n=11,939)	2.87 (4.68)		3.07 (4.46)		1102 (9.23)			8059 (67.50)		2113 (17.70)		665 (5.57)	
	Quintile 4 (n=11,937)	3.47 (4.42)		3.78 (4.41)		948 (7.94)			7446 (62.38)		2669 (22.36)		874 (7.32)	
	Quintile 5: highest usage (n=11,936)	4.84 (4.84)		5.33 (4.75)		554 (4.64)			5876 (49.23)		3917 (32.82)		1589 (13.31)	

^a^Average weight loss in kilograms and percentage of starting body weight were calculated using last observation carried forward.

^b^Proportion of the sample within each category of weight loss: weight gain, 0 to less than 5% of starting body weight lost, 5% to less than 10% of starting body weight lost, and greater than or equal to 10% of starting body weight lost.

### Predictors of Weight Loss

On the basis of multiple linear regression, member characteristics (sex, age, socioeconomic status, starting BMI) and program characteristics (duration of membership and usage of the platform per day) accounted for 29.4% of the variance associated with the percentage total weight loss (F_6,58434_=920.01; *P*<.001). All predictors were significant, with platform usage being the strongest predictor of weight loss (β=.263; *P*<.001) and higher usage per day being associated with greater weight loss. Sex (β=–.086; *P*<.001) and starting BMI (β=.062; *P*<.001) were the next 2 strongest predictors of percentage body weight lost, with men and those with a higher starting BMI losing more weight.

### Sensitivity Analysis

Starters, by definition, did not have a weight entered into the platform at 12 weeks or more; therefore, we used baseline observation to carry forward their assumed weight loss to zero. The average weight loss for stayers was 4.9 kg. Using the baseline-observation-carried-forward approach, we assumed the average weight loss for all members was 1.8 kg. Multiple linear regression showed similar results to those of the primary analysis. The model accounted for 31.5% of the variance associated with percentage total weight loss, and platform usage was the strongest predictor of weight loss (β=.272; *P*<.001).

## Discussion

### Principal Results

This study examined the data generated through the CSIRO Total Wellbeing Diet Online, a commercial weight loss program, to determine its reach and effectiveness over its first 5 years of use. This analysis included usage data from almost all (59,686/61,164, 97.58%) of the individuals who had signed up between October 2014 to September 2019. The results indicated that the average weight loss across all members was 3.1% of their starting body weight but was considerably higher (5.3% of starting body weight) in the 22,658 members who completed the full 12-week program or more (37.96% of all members analyzed). This magnitude of weight loss was consistent with similar programs [17,19] and greater than that reported for some other web-based commercial programs [10]. However, it was less than that of commercial programs that use a more intensive, one-to-one consultation model [28].

Our finding that almost 1 in 2 members who completed the CSIRO Total Wellbeing Diet Online program lost 5% or more of their starting body weight has considerable public health relevance, as a loss of 5% of body weight is considered clinically significant in relation to reductions in risk of comorbidities [29]. This result is also similar to the findings of a 6-month German-based commercial weight loss program, although the evaluation of this program used a much smaller sample (N=479) [30]. Furthermore, overweight and obese status are related to the development of several chronic conditions, which place a huge burden on the health care system [1]. This study was also able to demonstrate that about 40% of members with obesity who stayed on the program were able to shift down a weight status category, with about 24% no longer classified as obese when they finished the program. Moreover, approximately 25% who were classified as overweight at the start of the program achieved a normal weight at the completion of the program. The increased risk of morbidity and mortality from many chronic conditions means programs that can shift people out of the higher-risk, obese weight status category can confer significant potential gains in health and quality of life for these individuals [31]. If the results demonstrated here can be achieved at an even greater scale, the potential social and economic benefits will be extremely significant.

One of the most important findings from this study was that members who were living in the most disadvantaged areas were one of the most successful groups on the program based on percentage of total weight loss. About 10% of members were living in areas considered to be among the most disadvantaged in Australia, and their average weight loss was 5.8% of starting body weight, and about 53% lost 5% percent or more of their starting body weight. Although this may be partly attributable to the higher starting BMI in this group, it is nevertheless an encouraging finding given that individuals of lower socioeconomic status are at increased risk of weight gain and obesity [32,33] but tend to have lower rates of uptake and completion of weight loss programs [34]. There are many unique challenges that have been cited for disadvantaged groups, including literacy, insufficient time, and lack of motivation [32]. It has also been suggested that individuals with lower income are less likely to recognize their unhealthy weight and therefore less likely to attempt weight loss than are higher income individuals [35]. It is likely that motivations for joining a weight loss program may vary across groups of differing socioeconomic status [32]. Overall, the CSIRO Total Wellbeing Diet Online program appears to be an effective weight loss program for individuals in lower socioeconomic groups, representing a simple, evidence-based, and effective intervention to address the public health challenge of being overweight or obese.

### Platform Usage and Weight Loss

Participant adherence is known to be a primary determinant of program success [36], and this study, like others [11,37], showed that higher engagement with an online platform and more frequent usage of program features was associated with greater early weight loss and greater total weight loss. Members with the highest platform usage lost twice as much weight than did those with the lowest levels of usage. A review of technology-driven weight loss interventions suggests that self-monitoring, feedback and communication, social support, use of a structured program, and use of an individually tailored program are factors that could facilitate weight loss [38]. Opportunity for self-monitoring through entries in a diary [30,37,39,40] and engagement with social forums have been shown to be associated with greater weight loss and weight loss maintenance in web-based interventions [11,37,41]. On this program, members with the highest platform usage were using the food diary to record dietary intake about twice per day. They were also monitoring their body weight more closely by entering a weight value into the platform once per week. Consistent self-monitoring of weight is crucial to successful weight loss and weight loss maintenance [42] and is central to behavioral weight loss programs [43].

The specific features of a program are important and can influence engagement and weight loss. Different technologies may lend themselves to different features. The focus of this study was on a web-based program, but commercial weight loss programs delivered through mobile phone apps are also common. One of the most popular publicly available apps for weight loss is Noom Coach, with tens of millions of installs worldwide. An evaluation of Noom Coach used data from a very select group who had recorded their data at least twice per month for 6 consecutive months (n=36,000). In this highly motivated subsample, who were engaged and used the app in this way, 23% of users achieved a weight loss of 10% or more over the 6-month period examined [44]. For the CSIRO Total Wellbeing Diet Online, 20% (4532/22,658) of an engaged subsample (that is the highest platform usage users who stayed on the program for at least 3 months) achieved a weight loss of 10% or more. However, it would be interesting to see a larger-scale study with a greater proportion of total Noom users to better understand the weight loss a typical user can expect when embarking on the program. Mobile phone apps can offer different features, such as push notifications and real time information, which may provide additional support that is not available in a web-based program. An increasing number of technologies are being used in parallel, and programs are available across multiple platforms to offer users greater flexibility and choice. As the complexity of program delivery increases, it is vital that robust evaluations are conducted to determine the elements of programs, the components of their delivery, and the combinations of these that are associated with successful weight loss.

Achieving sufficient engagement and user retention is a challenge. For commercial weight loss programs, high attrition rates of up to 70% are common [28,45], and nonusage attrition is also high on web-based weight loss programs [46]. It is important to look at both program attrition and attrition in terms of technology usage because it is possible that people stop using the platform features before they formally drop out [46]. Platform usage could be thought of as the equivalent of treatment dose in medical studies, and so, while the optimal dose for web-based interventions is unclear, strategies to improve engagement need to be determined. Engagement and retention on a program might be related to user characteristics such as starting weight, as people who are heavier have more weight to lose to reach their weight loss goal and therefore are willing to stay on a program for longer [28]. In this study, starters and stayers had a similar starting BMI, but stayers might have been more intrinsically motivated to adhere to the program and achieve weight loss. A greater understanding of user behaviors that predict engagement and whether this varies for different user groups is needed to develop strategies to increase engagement and drive more successful weight loss outcomes. For example, posting a profile picture has been shown to predict higher engagement with an online weight loss program [47], and more frequent input of evening meal information is the most important behavior for weight loss in a commercial weight loss app [48]. If more specific behaviors like these were recognized, then they could be incorporated and specifically promoted to help achieve higher levels of engagement and weight loss.

Rapid initial weight loss has been shown to predict greater longer-term weight loss [30,49], and while this study showed higher usage of the platform was associated with greater early weight loss, further exploration could determine specific behavioral patterns that support early success. Data were not available for members once they had dropped out (and thus our results represent the best-case scenario), and the reasons for drop-out and nonplatform usage are also worthy of further investigation. It is unknown how weight loss is maintained once membership has ceased, but the achievement of long-term weight loss or weight loss maintenance following completion of the program is the true goal.

### Strengths and Limitations

A key strength of the current study is that a broad set of inclusion criteria was applied for analysis, and data from all members were used in reporting the weight loss results, which is not always the case for commercial programs [9,10,44]. Weight loss was conducted using self-reported values with the last weight entered into the platform being carried forward. As we split the sample into starters and stayers and presented the data separately, the weight losses reported for stayers are likely to be reflective of an “average” member that joins and finishes the program and not a selected subsample. Although this is a retrospective analysis, it represents a very large cohort of people who had chosen to join a commercial weight loss program and followed the program in their own everyday context. However, a limitation associated with this design is that the study included only the member characteristics that were collected as part of the registration process. Although a range of personal demographic data was included, other factors, such as past dieting history or motivation, were not available. Platform usage provided an indication of engagement, while viewing the menu plans and use of the food diary may relate to compliance with the eating plan. However, more objective and validated measures of dietary intake would better represent dietary program compliance.

### Conclusions

To our knowledge, this 5-year evaluation of the CSIRO Total Wellbeing Diet Online represents one of the most comprehensive evaluations of a commercially available, online weight loss program and has provided insights into predictors of weight loss success among members. Health care practitioners and individuals require evidenced-based weight management programs that have been shown to achieve meaningful weight loss. The findings suggest that this program is effective for weight loss, particularly for members who finish the program and are active in using the online platform and tools provided. Importantly, members with a higher starting BMI and those from more disadvantaged socioeconomic areas were among those who lost the most weight.

## Appendix

Multimedia Appendix 1 Mean (SD) starting body weight and body mass index of starters (n=37,028), stayers (n=22,658) and all members (n=59,686) at the time of joining the CSIRO Total Wellbeing Diet Online.

## Abbreviations

ANOVA: analysis of variance
CSIRO: Commonwealth Scientific and Industrial Research Organisation
RCT: randomized controlled trial
SEIFA: Socio-Economic Indexes for Areas
