# Supplementary material for: Weight Loss and Usage of an Online Commercial Weight Loss Program (the CSIRO Total Wellbeing Diet Online) Delivered in an Everyday Context: Five-Year Evaluation in a Community Cohort
Source: J Med Internet Res. 2021 Jun 7;23(6):e20981. doi: 10.2196/20981 (PMC8218211; doi:10.2196/20981)
Supplement: Multimedia Appendix 1 [file jmir_v23i6e20981_app1.docx]

Supplementary Table 1. Mean (SD) starting body weight and body mass index of starters (n=37028), stayers (n=22658) and all members (n=59686) at the time of joining the CSIRO Total Wellbeing Diet Online

| **Member characteristics** | | **Starters** | | **Stayers** | | **All members** | |
| --- | --- | --- | --- | --- | --- | --- | --- |
|  |  | **Starting weight** | **Starting BMI** | **Starting weight** | **Starting BMI** | **Starting weight** | **Starting BMI** |
|  | Total | 90.1 (20.2) | 32.2 (6.6) | 90.4 (18.8) | 32.2 (6.0) | 90.2 (19.7) | 32.2 (6.3) |
| **Sex** | Male | 105.4 (20.4) | 33.1 (5.9) | 104.3 (18.2) | 32.8 (5.5) | 104.9 (19.5) | 33.0 (5.8) |
|  | Female | 87.0 (18.6) | 32.0 (6.7) | 87.0 (17.3) | 32.0 (6.1) | 87.0 (18.2) | 32.0 (6.5) |
| **Age group (years)** | 18-30 | 90.0 (21.6) | 31.3 (6.5) | 89.1 (20.3) | 31.0 (6.1) | 89.7 (21.2) | 31.2 (6.4) |
|  | 31-50 | 90.6 (20.9) | 32.0 (6.7) | 91.5 (19.8) | 32.0 (6.2) | 91.0 (20.5) | 32.0 (6.5) |
|  | 51-70 | 89.7 (19.4) | 32.5 (6.5) | 90.0 (18.2) | 32.4 (5.9) | 89.8 (18.9) | 32.4 (6.3) |
|  | 71 + | 88.1 (18.6) | 32.0 (6.0) | 88.8 (16.7) | 32.2 (5.5) | 88.4 (17.8) | 32.1 (5.7) |
| **Starting BMI category** | Normal weight | 65.3 (6.9) | 23.4 (1.3) | 66.1 (6.7) | 23.6 (1.1) | 65.5 (6.8) | 23.4 (1.3) |
|  | Overweight | 77.8 (8.9) | 27.7 (1.4) | 78.1 (9.1) | 27.7 (1.4) | 77.9 (9.0) | 27.7 (1.4) |
|  | Obese | 101.0 (18.2) | 36.2 (5.6) | 100.3 (17.2) | 35.7 (5.2) | 100.8 (17.8) | 36.0 (5.5) |
|  | Class 1 | 91.0 (10.4) | 32.3 (1.4) | 91.4 (10.7) | 32.2 (1.4) | 91.2 (10.5) | 32.3 (1.4) |
|  | Class 2 | 103.7 (11.8) | 37.1 (1.4) | 104.0 (11.8) | 37.1 (1.4) | 103.8 (11.8) | 37.1 (1.4) |
|  | Class 3 | 124.7 (19.8) | 45.3 (5.7) | 123.7 (17.7) | 44.8 (5.1) | 124.3 (19.1) | 45.1 (5.5) |
|  | Missing/invalid | 91.7 (21.5) | - | 88.0 (19.9) | - | 91.6 (21.4) | - |
| **Socioeconomic Status** | 1 Lowest | 94.0 (21.4) | 34.0 (7.3) | 94.1 (19.7) | 33.7 (6.4) | 94.1 (20.8) | 33.9 (6.9) |
|  | 2 | 91.7 (20.4) | 32.9 (6.7) | 92.2 (19.4) | 33.0 (6.4) | 91.9 (20.0) | 33.0 (6.6) |
|  | 3 | 91.3 (20.5) | 32.6 (6.6) | 91.9 (19.4) | 32.6 (6.0) | 91.5 (20.1) | 32.6 (6.4) |
|  | 4 | 89.7 (19.9) | 32.0 (6.4) | 90.2 (18.4) | 32.0 (5.9) | 89.9 (19.3) | 32.0 (6.2) |
|  | 5 Highest | 87.7 (19.2) | 31.1 (6.1) | 87.7 (17.8) | 31.1 (5.5) | 87.7 (18.7) | 31.1 (5.9) |
|  | Unknown | 88.7 (21.3) | 31.5 (6.3) | 92.0 (20.9) | 32.4 (6.3) | 89.6 (21.2) | 31.8 (6.3) |
